# Integrated transcriptome and metabolome analysis reveal the sesquiterpenoid biosynthesis mechanism of *Atractylodes chinensis* under drought stress

**DOI:** 10.3389/fpls.2025.1751860

**Published:** 2026-01-26

**Authors:** Ying Wu, Yao Lu, Qiuju Ye, Bingqian Jin, Boqian Jiang, Dan Wang, Haibo Yin

**Affiliations:** School of Pharmacy, Liaoning University of Traditional Chinese Medicine, Dalian, Liaoning, China

**Keywords:** *Atractylodes chinensis*, drought stress, metabolomics, sesquiterpenoids, transcriptomics

## Abstract

**Introduction:**

As a traditional Chinese medicine abundant in sesquiterpenoids, *Atractylodes chinensis* has various pharmacological activities. Nevertheless, the synthesis and metabolism mechanisms of its sesquiterpenoids under drought stress are still not fully elucidated.

**Methods:**

Therefore, this study investigated changes in sesquiterpenoid component contents, gene expression profiles, and metabolite accumulation of *A. chinensis* under drought stress.

**Results:**

Results showed that the moderate drought stress (MDS) significantly increased the contents of atractylodin, β-eudesmol, and atractylenolide I. Compared with the control group (CK), 10,528, 9,755, and 10,562 differentially expressed genes (DEGs) were identified in the light drought stress (LDS), MDS, and severe drought stress (SDS) groups, respectively. These DEGs are involved in plant–pathogen interaction, plant hormone signal transduction, plant MAPK signal transduction, and starch and sucrose metabolism. Metabolic analysis detected 2,101, 2,112, and 2,144 differentially accumulated metabolites (DAMs) in the LDS, MDS, and SDS groups, including atractylodin, β-eudesmol, and atractylenolide I. These DAMs are primarily enriched in three pathways: “ABC transporters”, “D-amino acid metabolism”, and “aminoacyl-tRNA biosynthesis”. Furthermore, we screened and characterized the expression patterns of DEGs and accumulation levels of DAMs involved in the sesquiterpenoid biosynthesis pathway. Notably, the genes *TRINITY_DN12874_c1_g1*, *TRINITY_DN114406_c0_g1*, *TRINITY_DN2331_c0_g2*, *TRINITY_DN7401_c0_g1*, *TRINITY_DN11676_c0_g1*, along with the compound Germacra-1(10),4,11(13)-trien-12-ol, are speculated to be key genes and critical metabolite responding to drought stress, respectively.

**Discussion:**

These findings enhance our understanding of the mechanisms by which drought stress modulates the sesquiterpenoid biosynthesis pathways in *A. chinensis*.

## Introduction

**Figure 1:**
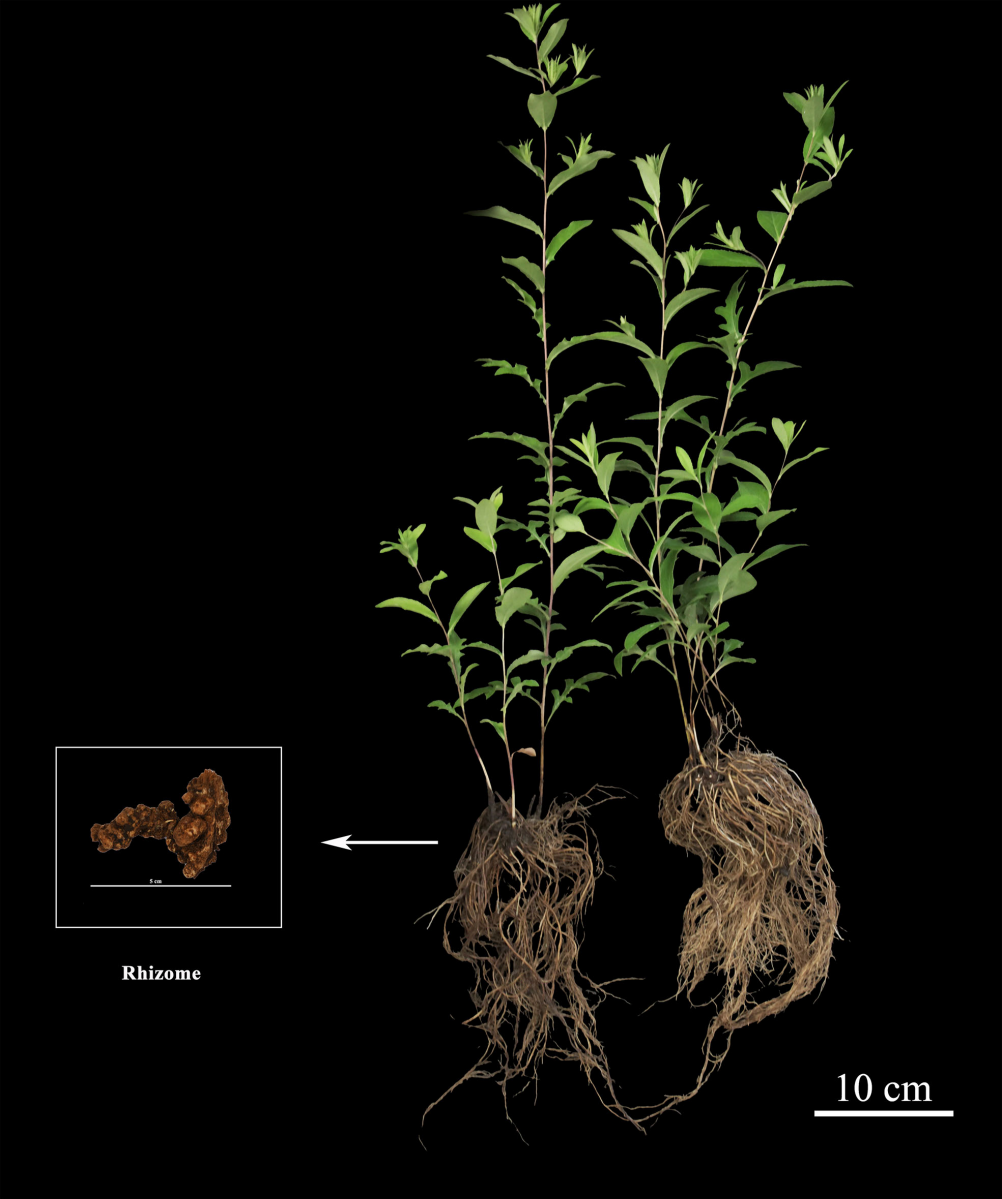
*Atractylodes chinensis* (DC.) Koidz.

*Atractylodes chinensis* (DC.) Koidz., a perennial herbaceous species within the genus *Atractylodes* of the Asteraceae family, holds significant medicinal value due to its dried rhizomes (**Figure 1**), which possess the effects of drying dampness to invigorate the spleen, dispelling wind and cold, and improving eyesight, which have long been utilized as a core medicinal material in traditional East Asian medicine (e.g., Traditional Chinese Medicine). Ecologically, this species exhibits a preference for specific habitats, typically thriving in hillside grasslands, forest understory habitats, shrubby thickets, and rocky crevices. Geographically, its natural distribution spans multiple northern and northeastern regions of China, including Liaoning, Jilin, Hebei, and Inner Mongolia, among others (Zhang et al., 2021). The market demand for *Atractylodis Rhioma* remains substantial. Nevertheless, wild resources are severely deficient and thus unable to meet such demand, which has rendered artificial cultivation the predominant supply source in the market. With the vigorous advancement of large-scale cultivation practices, improving the quality of cultivated *A. chinensis* has emerged as an urgent priority critical to the sustainable development of traditional Chinese medicine. The medicinal efficacy of *A. chinensis* is largely attributed to its bioactive secondary metabolites, with sesquiterpenoids and polyenes identified as the primary bioactive component classes (Zhuang et al., 2022**;**He et al., 2011). Among these, sesquiterpenoids exhibit notably high accumulation levels in its rhizomes, with key representative compounds including atractylodin, β-eudesmol, and atractylenolide I, which are widely recognized as quality markers for *A. chinensis* medicinal products (Chu et al., 2011). As pivotal determinants of the medicinal quality and therapeutic potential, these sesquiterpenoids have been experimentally validated to exert a broad spectrum of biological activities, encompassing anti-inflammatory, anti-tumor, neuroprotective, hepatoprotective, antibacterial, and antiviral effects (Liu et al., 2020**;**Sun et al., 2022**;**Pang et al., 2021). To contextualize the biosynthesis of these critical sesquiterpenoids, it is essential to consider the universal terpenoid backbone biosynthetic networks in plants, which primarily rely on two evolutionarily conserved core pathways. The first is the mevalonate (MVA) pathway, which is localized in the cytoplasm; the second is the methylerythritol phosphate (MEP) pathway, which is exclusively situated in plastids (Vranová et al., 2013**;**Buhaescu and Izzedine, 2007). For the MVA pathway specifically, its primary metabolic output consists of terpenoid precursors (Roberts, 2007**;**Lange and Ahkami, 2013), more precisely, cytoplasmic isopentenyl pyrophosphate (IPP) serves as a direct precursor for the synthesis of farnesyl pyrophosphate (FPP)—a key intermediate that is not only critical for sesquiterpenoid biosynthesis but also functions as a universal precursor for the entire class of terpenoid compounds (Zhao et al., 2015). Following its formation, FPP is further catalyzed by specific enzymes to diverge into two distinct biosynthetic branches: one dedicated to sesquiterpenoid metabolism and the other to triterpenoid metabolism.

During their growth and development, plants frequently encounter stresses of varying intensities. Upon exposure to stress, plants initiate a suite of self-regulatory mechanisms—encompassing stress sensing, signal transduction, transcriptional activation, translational regulation, and post-translational protein modification—to modulate the biosynthetic pathways of metabolites. These regulatory processes are critical for sustaining normal growth and development under adverse conditions (Zhang et al., 2022). Among abiotic stresses, drought stress is recognized as the most impactful, exerting profound effects on plant growth, development, and metabolic homeostasis (Razi and Muneer, 2021). With the prolongation of drought duration, plants activate a series of intricate regulatory networks to fine-tune the expression of stress including responsive protective genes, thereby enhancing their ability to adapt to or tolerate drought stress (Zhang S. et al., 2024). Secondary metabolites play a pivotal role in mediating plant responses to abiotic stress. For instance, in tobacco, the up-regulation of key metabolites (e.g., chlorogenic acid, rutin, and cedarine) and their corresponding biosynthetic genes strengthens antioxidant capacity, which in turn mitigates oxidative damage induced by drought stress (Yin et al., 2025). Drought stress increases the content of abscisic acid (ABA), thereby activating the expression of *AREB1*, regulating the expression of *TAS14*, *GSH-Px-1*, and heat shock proteins, and ultimately improving the drought resistance of tomatoes (Liu et al., 2023). Additionally, genes such as *WRKY22*, *WRKY52*,*GID1*, *JAR1*, and *DELLA* have been implicated in both phenylpropanoid and flavonoid biosynthesis, as well as plant hormone signal transduction. Notably, these genes exhibit dual functional roles: they not only mediate responses to drought stress but also regulate the accumulation of tanshinone (Zhou et al., 2024).

MDS has been shown to promote the accumulation of secondary metabolites, including terpenoids, flavonoids, and alkaloids (Li et al., 2025**;**Zhou et al., 2025a; Kleiber et al., 2017; Huang et al., 2024; Zhou et al., 2025b). Specifically, drought stress exerts multiple regulatory effects on plant metabolic pathways: it up-regulates the expression of key genes in metabolic networks, thereby facilitating the biosynthesis and accumulation of amino acids and alkaloids (Zhang S. Y. et al., 2024); it activates the expression of genes encoding antioxidant enzymes, which in turn drives flavonoid biosynthesis (Zhang et al., 2023); it modulates the terpenoid backbone biosynthesis pathway to supply precursors for both primary and secondary metabolism (Wang et al., 2023); and it regulates the expression of sesquiterpenoid-related genes, increasing the expression levels of *HMGR* and *Ovtps2*, while decreasing those of *FPPS* and *Ovtps6*—thereby altering the biosynthesis and accumulation of sesquiterpenoid components (Morshedloo et al., 2017). MDS has been reported to enhance the contents of primary metabolites (e.g., soluble sugars and crude proteins) and elevate the activity of antioxidant enzymes in *A. chinensis* (Ma et al., 2024). However, the accumulation patterns and underlying metabolic mechanisms of sesquiterpenoids under drought stress remain inadequately elucidated. In this study, we integrated transcriptomic and metabolomic data to analyze the biosynthetic mechanism of sesquiterpenoids in *A. chinensis* under drought stress. Specifically, we aimed to identify the key genes and critical metabolites, and further clarify the effects of drought stress on changes in the content of secondary metabolites and their underlying mechanisms, which is of great significance for improving the quality of *Atractylodis Rhioma.*

## Materials and methods

### Plant material and stress treatment

Two-year-old *A. chinensis* seedlings were planted in the Medicinal Botanical Garden of Liaoning University of Traditional Chinese Medicine. Seedlings with uniform growth were selected for pot cultivation, with 3 seedlings per pot. Four treatment groups were established, where soil water contents were maintained at 75%–80%, 55%–60%, 35%–40%, and 15%–20% of the maximum field water capacity (26%)—corresponding to the CK, LDS, MDS, and SDS, respectively. A total of 432 seedlings were used with 108 seedlings allocated to each group. The weighing and water supplementation method was adopted to maintain soil water content at the preset gradients. Stress treatment was continued for 143 days, from the seedling stage (May 28, 2025) to the fruiting stage (October 17, 2025). The rhizomes of *A. chinensis* were immediately frozen in liquid nitrogen and stored at -80 °C for subsequent total RNA extraction, sesquiterpenoid content determination, and multi-omics analysis. Three biological replicates were set for each treatment group in all aforementioned experiments.

### Analysis of sesquiterpenoid content

Approximately 0.2 g rhizome powder was weighed into a stoppered conical flask and ultrasonically extracted with 10 mL of methanol (analytical grade) at 400 W and 25 °C for 1 h (ultrasonic cleaner model: KQ5200DB, Kunshan Ultrasonic Instrument Co., Ltd., Kunshan, China). The extract was filtered through a 0.22 μm organic phase filter membrane. HPLC analysis was performed using an Agilent 1260 system equipped with a reversed-phase C18 column (Dikma Diamonsil Plus, 5 μm, 250 mm × 4.6 mm, Beijing, China). The detection parameters for sesquiterpenoid content were set as follows: mobile phase, water/acetonitrile (2:8, v/v); flow rate, 0.8 mL·min^−^¹; column temperature, 30 °C; detection wavelength, 208 nm; injection volume, 10 μL.Individual standard compounds (atractylodin, β-eudesmol, and atractylenolide I, purity ≥ 98%, Shanghai Yuanye Biotechnology Co., Ltd., Shanghai, China) were used to establish standard curves for quantifying the contents of the three sesquiterpenoids.

### RNA sample preparation and high-throughput sequencing

Total RNA was extracted using TRIzol reagent (Invitrogen, Carlsbad, CA, USA) according to the manufacturer’s protocol. RNA quality was evaluated using 1% agarose gel electrophoresis (to assess RNA integrity) and an Agilent 2100 Bioanalyzer (Agilent Technologies, Santa Clara, CA, USA). After total RNA extraction, oligo magnetic beads were used to enrich eukaryotic mRNA. The enriched mRNA was randomly fragmented by adding mRNA-specific fragmentation buffer. First-strand and second-strand cDNA were synthesized using the fragmented mRNA as a template, followed by cDNA purification. The purified double-stranded cDNA was subjected to end repair, A-tailing, and sequencing adapter ligation. Fragment size selection was performed using AM Pure XP beads (Beckman Coulter, Brea, CA, USA) to enrich the cDNA library, which was then sequenced on an Illumina Novaseq 6000 platform (Illumina, San Diego, CA, USA).

Transcriptome assembly was performed using Trinity v2.14.0 with default parameters. The FPKM (Fragments Per Kilobase of transcript per Million mapped reads) values were calculated using RSEM v1.2.19 to quantify gene expression abundance. DESeq2 v1.30.1 was used for pairwise differential expression analysis between the CK and each drought stress groups. Genes with a false discovery rate (FDR) < 0.01 and absolute fold change (|fold change|) ≥ 2 were defined as DEGs.

### Quantitative real-time reverse transcription PCR analysis

The expression patterns of DEGs were verified by qRT-PCR. cDNA templates of each sample were obtained using HiScript III RT SuperMix for qPCR (+gDNA wiper) (Vazyme, Nanjing, China) according to the manufacturer’s instructions. qRT-PCR was performed on the ABI 7500 Fast Real-Time System (Applied Biosystems, New York, USA) with TransStart Top Green qPCR Supermix (TransGen Biotech, Beijing, China) to verify the transcriptional levels of 15 selected DEGs. All samples were evaluated with three biological replicates and three technical replicates. The 2^−ΔΔCt^ method was used to calculate the relative expression levels of DEGs.

### Metabolite extraction, measurement, and analysis

Rhizomes of *A. chinensis* were freeze-dried, and 50 mg of dried powder was weighed per sample. Metabolites were extracted by adding 1000 μL of extraction solution (analytical grade methanol:acetonitrile:water = 1:2:1, v/v/v) to the powder, followed by vortexing for 30 s, and sonication at 400 W and 25 °C for 10 min. The mixture was incubated at -20 °C for 1 h and then centrifuged at 12,000 rpm at 4 °C for 15 min. The supernatant was filtered through a 0.22 μm organic phase filter membrane for subsequent analysis. All samples were analyzed with three biological replicates.

Metabolomic analysis was performed using a UPLC-MS/MS system (Waters Corporation, Milford, MA, USA). The chromatographic column employed was a Waters ACQUITY UPLC HSS T3 C18 column (particle size: 1.8 μm, column dimension: 2.1 mm × 100 mm). The mobile phases were solvent A (aqueous ammonium acetate containing 0.1% formic acid) and solvent B (acetonitrile containing 0.1% formic acid). The elution gradient was as follows: 0 min, 98% A/2% B; 1.5 min, 98% A/2% B; 5.0 min, 50% A/50% B; 9.0 min, 2% A/98% B; 10.0 min, 2% A/98% B; 11.0 min, 98% A/2% B; 14.0 min, 98% A/2% B. The flow rate was 350 μL·min^−^¹, the column temperature was set at 40 °C, and the injection volume was 2 μL. For MS/MS detection, the electrospray ionization (ESI) source temperature was set to 550 °C. The voltage of the QTRAP^®^6500+ mass spectrometer was 5500 V (positive ion mode) and -4500 V (negative ion mode). Ion source gas I (GSI), gas II (GSII), and curtain gas (CUR) were set to 50, 55, and 35 psi, respectively, and the collision-induced dissociation (CID) parameter was set to medium. The original peak area data were normalized by the total peak area prior to subsequent analysis. Principal component analysis (PCA) and Spearman’s rank correlation analysis were used to evaluate the repeatability of intra-group samples and quality control samples. DAMs were screened by combining log_2_FC, p-value, and variable importance in projection (VIP) from the orthogonal partial least squares-discriminant analysis (OPLS-DA) model, with screening criteria: |log_2_FC| > 1, p < 0.05, and VIP > 1.

## Results

### Accumulation of sesquiterpenoids in *A. chinensis* under drought stress

**Figure 2:**
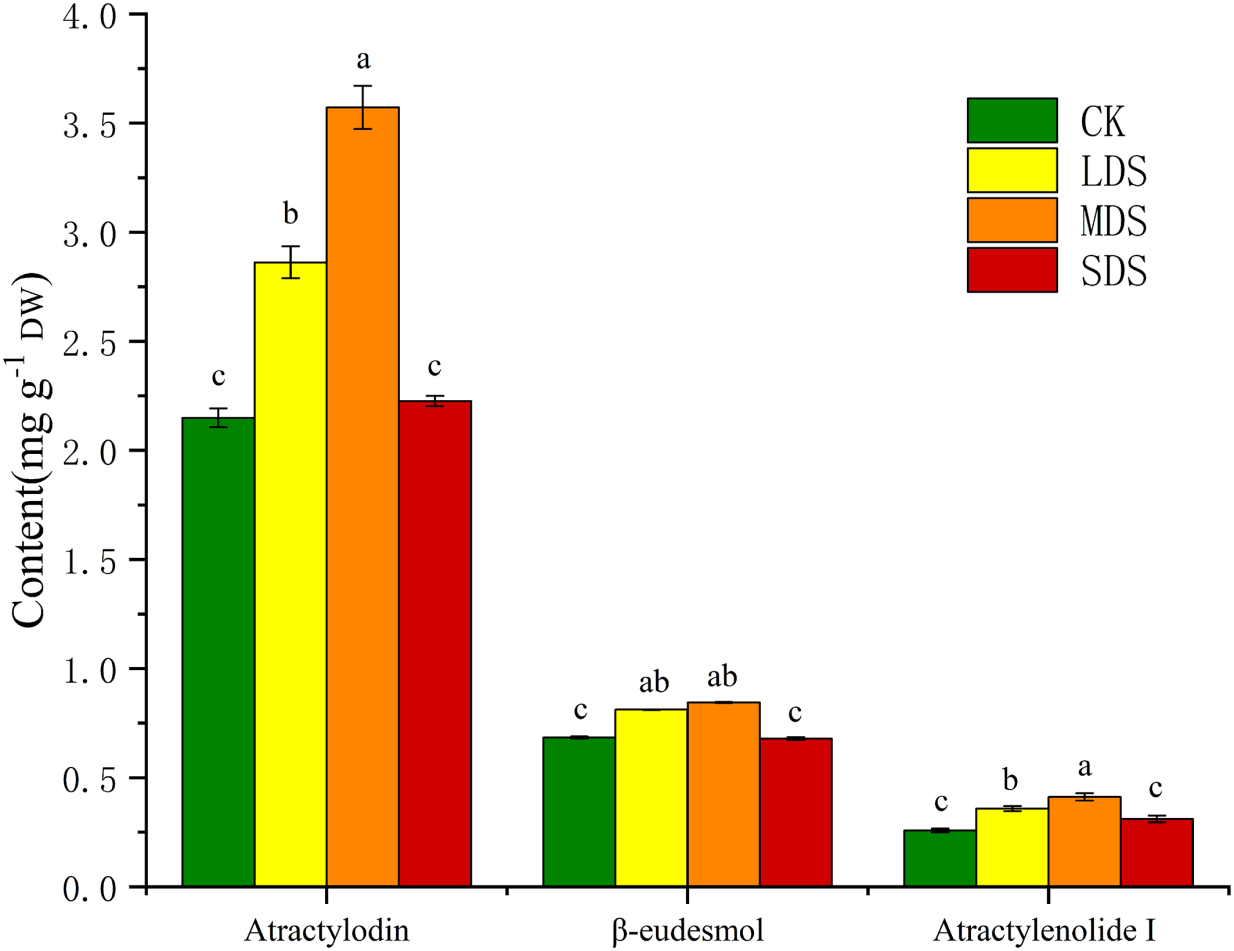
Influence of different drought stress levels on atractylodin, β-eudesmol, and atractylenolide I. Error bars indicate SD (*n = 6*).

The contents of atractylodin, β-eudesmol, and atractylenolide I in *A. chinensis* treated with different stress levels were determined by HPLC (**Figure 2**). The sesquiterpenoid content reached the maximum under MDS and then declined. HPLC results revealed that light and moderate drought significantly promotes the accumulation of sesquiterpenoids in *A. chinensis*.

### Transcriptome and DEGs analysis

A total of 79.19 Gb of data were obtained, each sample’s Clean Data reaching 6.21 Gb and the Q30 base percentage being 95.35% or higher. After transcriptome assembly, 99,919 unigenes were generated. For the functional annotation of unigenes, 45,543 unigenes were successfully annotated in at least one of the 8 public databases (**Table 1**). To gain a deeper understanding of the molecular mechanism of *A. chinensis* in responding to drought stress, DEGs were identified. Compared to the CK, a total of 10,528 unigenes exhibited differential expression in response to LDS, with 4,497 up-regulated genes and 6,031 down-regulated genes (**Supplementary Table S1**). Similarly, in MDS group, 9,755 unigenes showed differential expression, with 3,890 up-regulated genes and 5,865 down-regulated genes (**Supplementary Table S2**). SDS resulted in the differential expression of 10,562 unigenes, with 4,908 up-regulated genes and 5,654 down-regulated genes (**Supplementary Table S3**). Notably, the highest number of DEGs (10,562) and the largest number of up-regulated genes (4,908) appeared in the CK vs SDS group. However, the largest number of down-regulated genes (6,031) was found in the CK vs LDS group.

**Table 1:** Functional annotation of unigenes in different databases.

Database	Exp_Unigene number (Percent)
COG	8381 (0.0839)
GO	35389 (0.3542)
KEGG	27644 (0.2767)
KOG	22625 (0.2264)
Pfam	24238 (0.2426)
Swiss-Prot	25663 (0.2568)
eggNOG	33190 (0.3322)
NR	43423 (0.4346)
All_annotated	45543 (0.4558)
Total Unigene	99919 (1)

**Figure 3:**
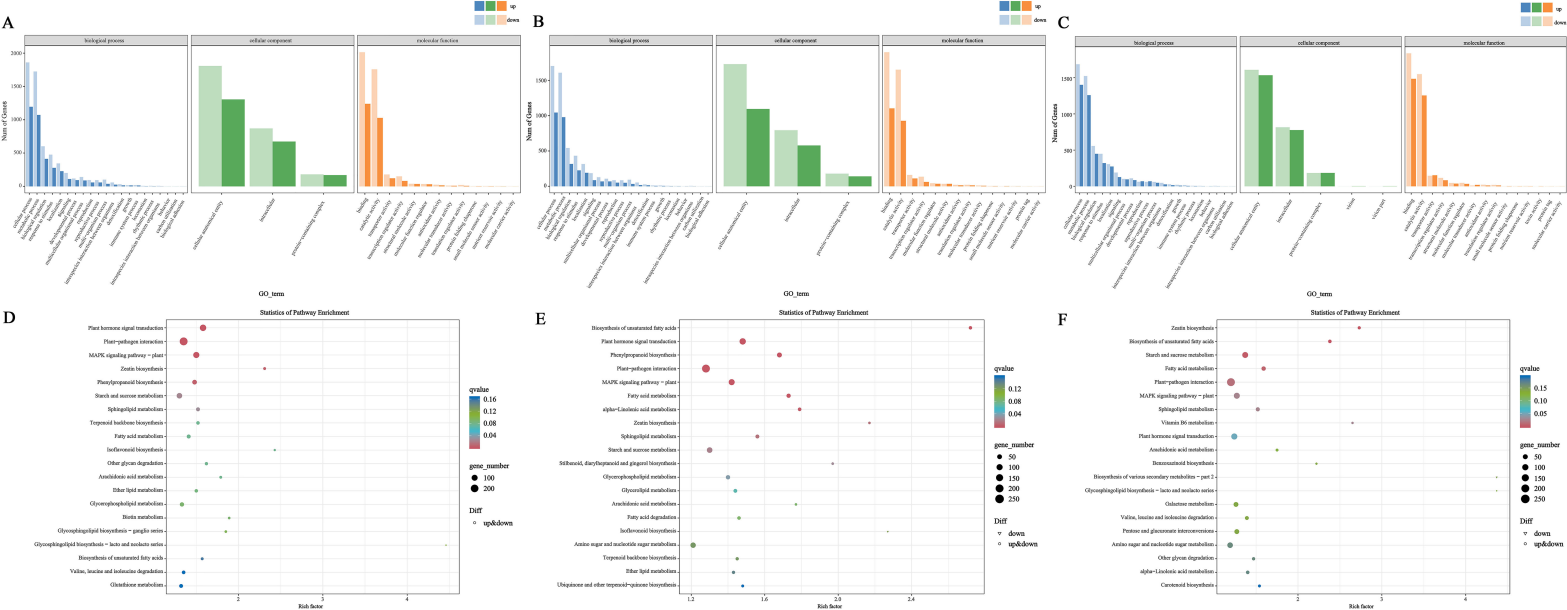
Functional enrichment analysis of DGEs. **(A)** GO enrichment analysis of DEGs in CK vs LDS. **(B)** GO enrichment analysis of DEGs in CK vs MDS. **(C)** GO enrichment analysis of DEGs in CK vs SDS. **(D)** Top 20 KEGG pathway enrichment in CK vs LDS. **(E)** Top 20 KEGG pathway enrichment in CK vs MDS. **(F)** Top 20 KEGG pathway enrichment in CK vs SDS.

To explore the biological functions of DEGs, we performed GO and KEGG pathway enrichment analyses. For the DEGs in CK vs LDS (**Figure 3A**), “cellular process” was the most enriched term in biological process (BP), “cellular anatomical entity” was the most enriched in cellular component (CC), and “binding” was the most enriched in molecular function (MF). In CK vs MDS (**Figure 3B**), enriched GO terms were mainly concentrated in BP, and “binding” (MF, 3002) was the most enriched. In CK vs SDS (**Figure 3C**), enriched GO terms were also mainly concentrated in BP, and the most enriched term was “binding” (MF, 3321). DEGs were involved in 135 metabolic pathways after LDS (**Figure 3D**), 133 after MDS (**Figure 3E**), and 136 after SDS (**Figure 3F**). Among these, the top 4 metabolic pathways in all three groups were plant–pathogen interaction, plant hormone signal transduction, MAPK signaling pathway in plants, and starch and sucrose metabolism.

### QRT-PCR validation

**Figure 4:**
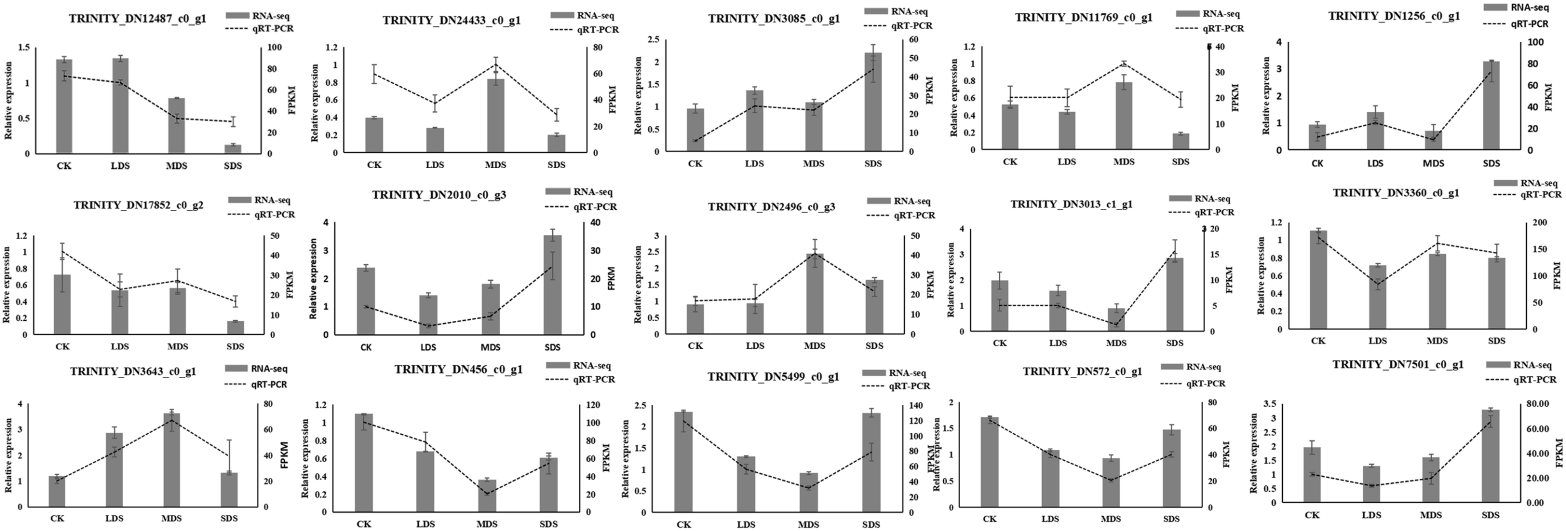
QRT-PCR validation of sesquiterpenoids biosynthesis-related genes in *A. chinensis* under drought stress. The line chart represents the gene expression levels of the qRT-PCR results, and the histogram represents FPKM values of sequencing results.

Through qRT-PCR analysis, the expression trends of the 15 selected DEGs involved in the sesquiterpenoid biosynthesis pathway were basically consistent with those in the transcriptome (**Figure 4**). Therefore, the transcriptome data can accurately reflect the expression profiles of genes related to sesquiterpenoid biosynthesis in *A. chinensis* under different drought stress treatment.

### Metabolome and DAMs analysis

**Figure 5:**
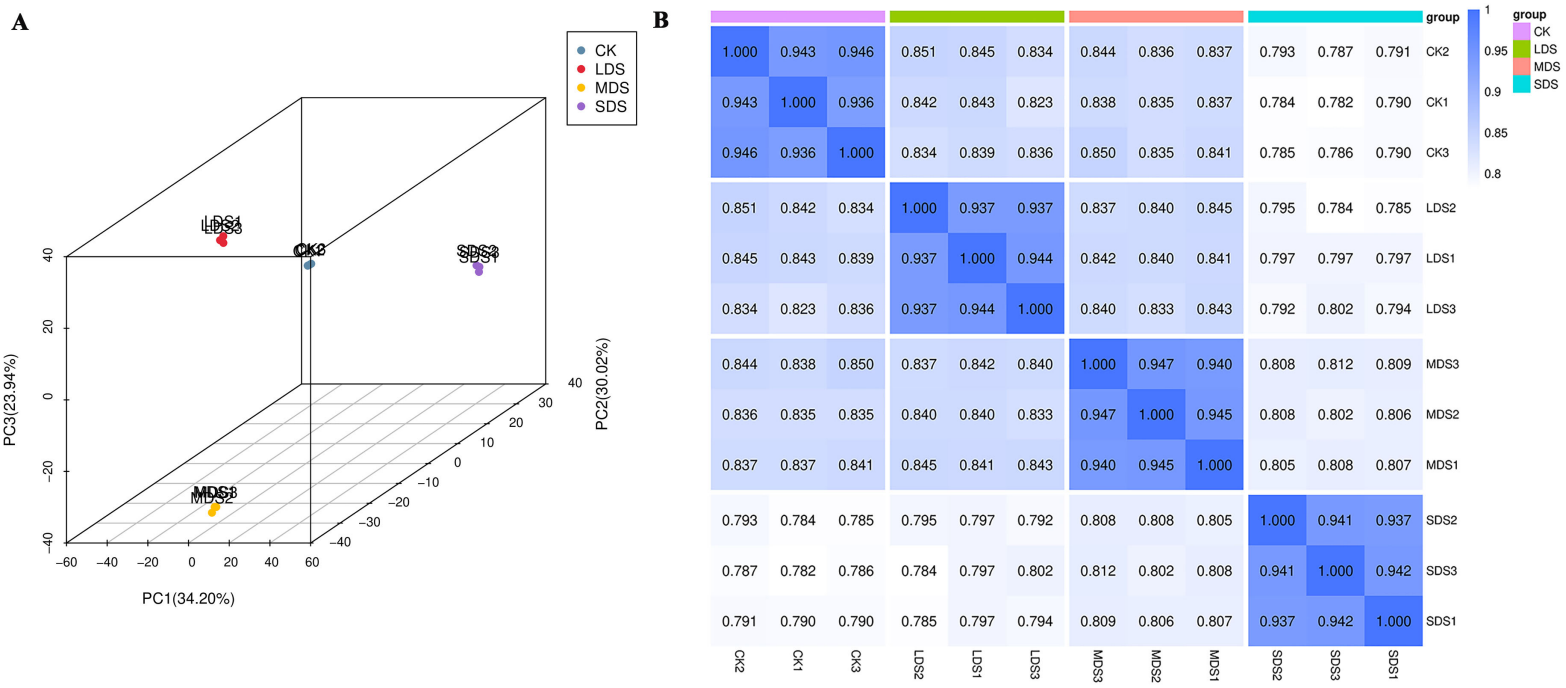
PCA analysis of all samples and correlation diagram between samples. **(A)** PCA Analysis of All Samples. The X-axis represents the first principal component, the Y-axis represents the second principal component, and the Z-axis represents the third principal component. The percentage on each axis indicates the contribution ratio of that principal component to the sample differences. **(B)** Correlation Diagram Between samples. Both the horizontal and vertical axes represent sample names, and the depth of color indicates the magnitude of the correlation coefficient *r*.

**Figure 6:**
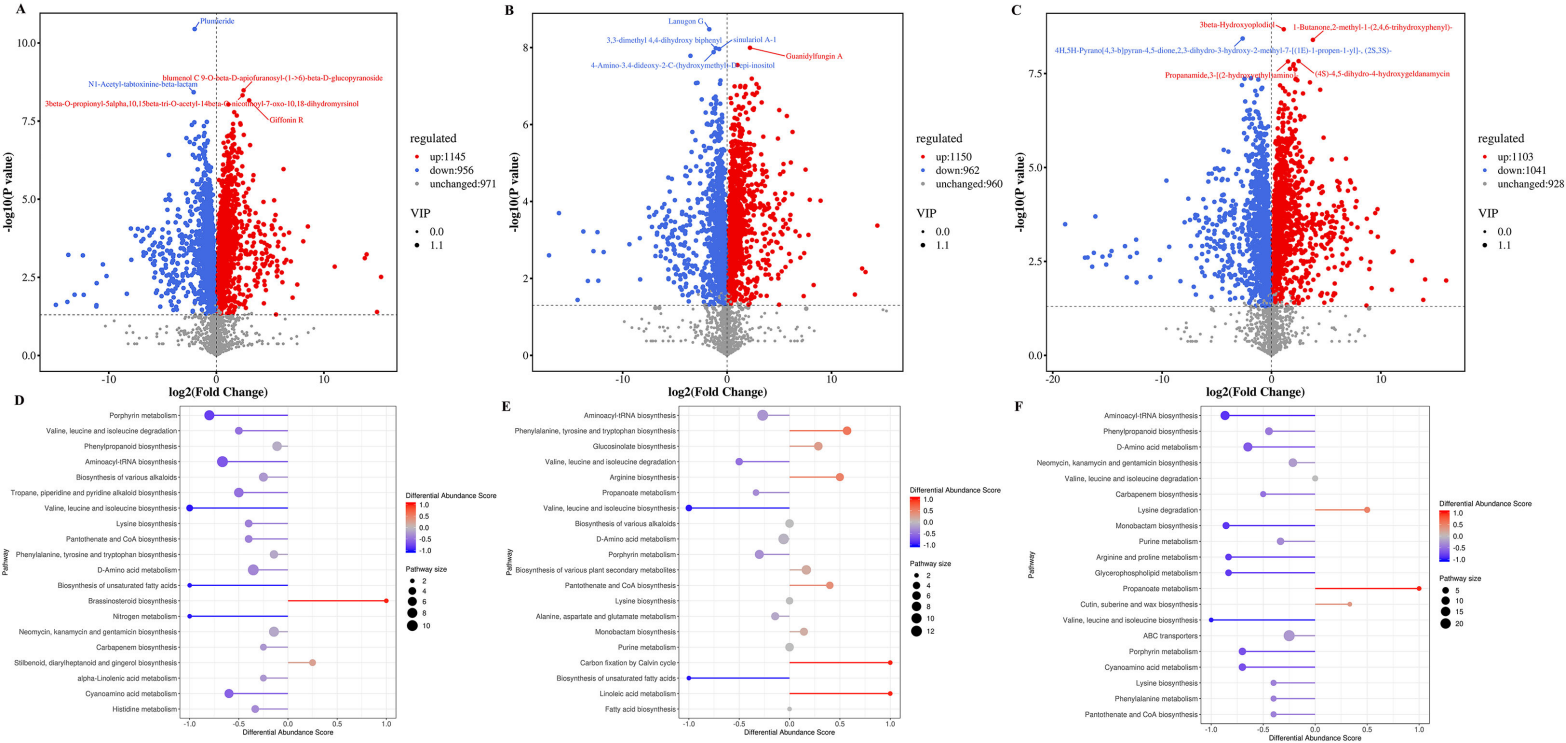
Analysis of differential metabolites under drought stress. **(A)** Volcano plot of DAMs in CK vs LDS. **(B)** Volcano plot of DAMs in CK vs MDS. **(C)** Volcano plot of DAMs in CK vs SDS. **(D)** Abundance score of DAMs in CK vs LDS. **(E)** Abundance score of DAMs in CK vs MDS. **(F)** Abundance score of DAMs in CK vs MDS.

To understand the changes in metabolites (**Supplementary Figure S1**) of *A. chinensis* under drought stress, a comprehensive metabolite analysis was conducted on samples, and DAMs were identified. The variations in metabolites were explored using PCA (**Figure 5A**) and Spearman Rank Correlation (**Figure 5B**), indicating the stability and replicability of the detection method. In addition, OPLS-DA score permutations were obtained, and evident differences were observed for CK vs LDS (R²Y=1, Q²Y=0.997), CK vs MDS (R²Y=1, Q²Y=0.996), and CK vs SDS (R²Y=1, Q²Y=0.997), demonstrating the suitability of the constructed model. In comparison to CK, 2,101 DAMs were found in the LDS group (**Figure 6A**; **Supplementary Table S4**), with 1,145 significantly up-regulated and 956 significantly down-regulated metabolites. A total of 2,112 metabolites were identified as DAMs in the MDS group (**Figure 6B**; **Supplementary Table S5**), consisting of 1,150 significantly up-regulated and 962 significantly down-regulated metabolites. In the SDS group (**Figure 6C**; **Supplementary Table S6**), a total of 2,144 DAMs were identified, among which 1,103 metabolites were significantly up-regulated and 1,041 were significantly down-regulated.

To reveal the pathways of DAMs, we performed a KEGG pathway enrichment analysis. In LDS and SDS groups (**Figures 6D, E**), the top five pathways were “ABC transporters”, “D-amino acid metabolism”, “aminoacyl-tRNA biosynthesis”, “porphyrin metabolism”, and “neomycin, kanamycin and gentamicin biosynthesis”. However, the top five pathways in MDS group were “ABC transporters”, “aminoacyl-tRNA biosynthesis”, “D-amino acid metabolism”, “biosynthesis of various plant secondary metabolites”, and “porphyrin metabolism” (**Figure 6F**).

### Correlation analysis of the transcriptome and metabolome of DEGs and DAMs involved in sesquiterpenoid biosynthesis pathways

**Figure 7:**
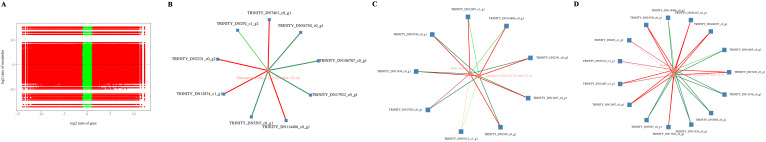
Exploration of the relationship between DEGs and DAMs using nine quadrant and co-expression network analysis. **(A)** Nine quadrant map of CK vs MDS. **(B)** Correlation network of DEGs and DAMs involved in the sesquiterpenoid and triterpenoid biosynthesis pathway in CK vs LDS. **(D)** Correlation network of DEGs and DAMs involved in the sesquiterpenoid and triterpenoid biosynthesis pathway in CK vs MDS. **(C)** Correlation network of DEGs and DAMs involved in the sesquiterpenoid and triterpenoid biosynthesis pathway in CK vs SDS.

To better understand the relationship between the metabolomic and transcriptomic profiles of different drought stress groups, a nine-quadrant analysis and a co-expression network analysis were performed. The nine-quadrant analysis was conducted to illustrate the difference ratios of DEGs and DAMs in the transcriptomic and metabolomic datasets of the “CK vs MDS” comparison, with a Pearson’s correlation coefficient > 0.8 and a P-value < 0.05 (**Figure 7A**). Genes and metabolites located in the third and seventh quadrants showed consistent differential expression patterns, indicating that the changes in metabolite accumulation might be positively regulated by genes. There were 24,255 and 34,290 genes involved in sesquiterpenoid biosynthesis-related responses in the third and seventh quadrants, respectively. In contrast, genes and metabolites located in the first and ninth quadrants showed opposite expression patterns, suggesting that the changes in metabolite accumulation might be negatively regulated by genes. A total of 26,911 and 30,846 genes related to sesquiterpenoid biosynthesis were found in the first and ninth quadrants, respectively.

Core DAMs and DEGs were identified based on the co-enrichment KEGG pathway analysis of the transcriptomic and metabolomic datasets. The “sesquiterpenoid and triterpenoid biosynthesis (ko00909)” pathway was enriched in CK vs LDS, CK vs MDS, and CK vs SDS. There were 9 genes and 1 metabolite co-enriched in CK vs LDS (**Figure 7B**), 9 genes and 2 metabolites in CK vs MDS (**Figure 7C**), and 15 genes and 2 metabolites in CK vs SDS (**Figure 7D**). The correlation network of DEGs and DAMs involved in sesquiterpenoid and triterpenoid biosynthesis revealed that Germacra-1(10),4,11(13)-trien-12-ol was either positively or negatively regulated by DEGs in CK vs LDS. In this correlation network, *TRINITY_DN12874_c1_g*1, *TRINITY_DN114406_c0_g1*, *TRINITY_DN2331_c0_g2*, and *TRINITY_DN7401_c0_g1* were positively correlated with Germacra-1(10),4,11(13)-trien-12-ol.

### Key DEGs and DAMs Co-enriched in sesquiterpenoid biosynthesis pathways

**Figure 8:**
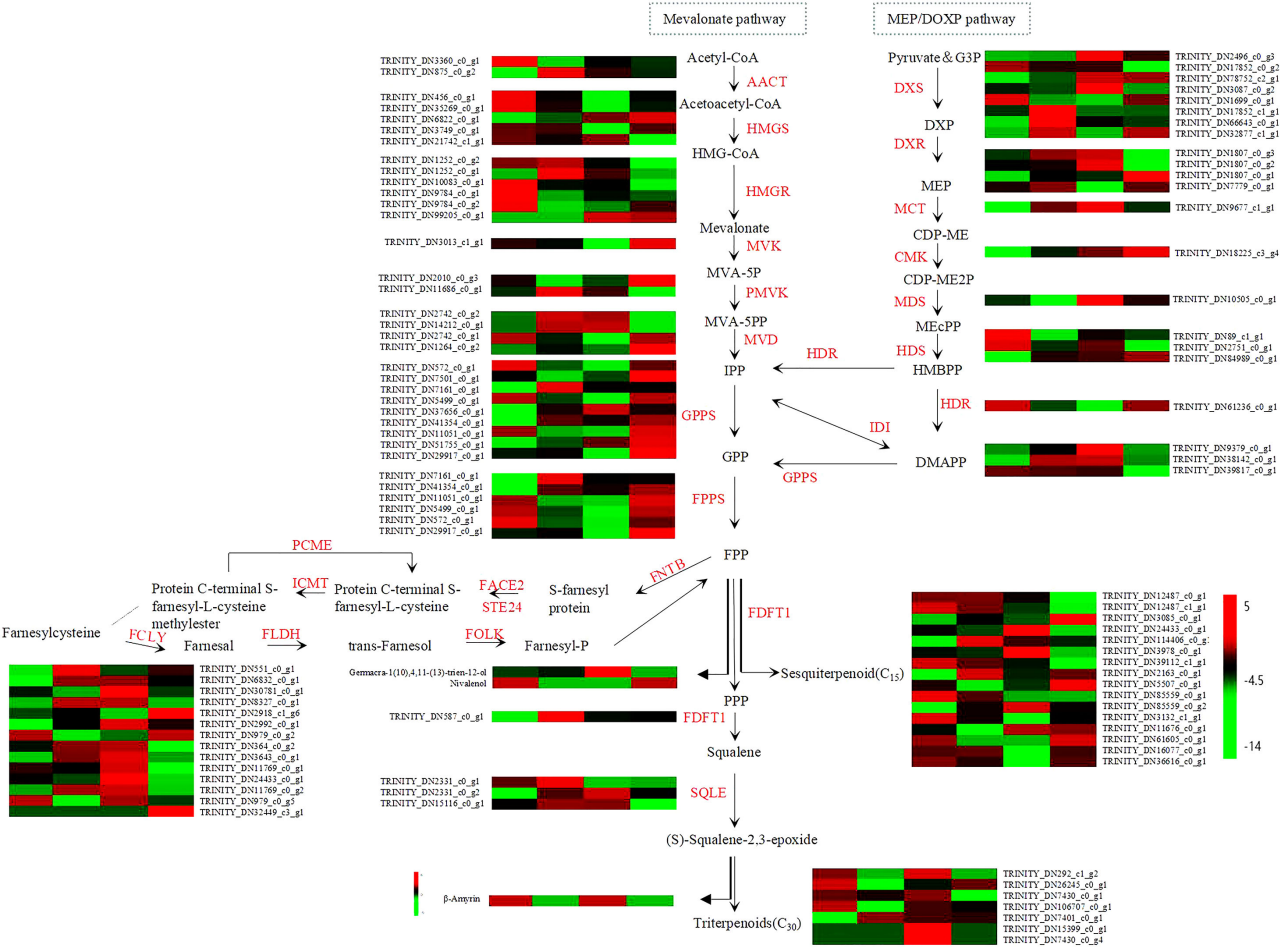
DEGs and DAMs involved in sesquiterpenoid and triterpenoid biosynthesis pathway (MVA, MEP/DOXP and downstream sesquiterpenoid biosynthesis pathways).Each colored block matrix represents a gene expression heatmap. Rows correspond to different genes, while columns represent the CK, LDS, MDS and SDS groups in sequence. The gene expression heatmap depicts the expression patterns of key enzyme genes: red indicates up-regulated gene expression, green indicates down-regulated gene expression, and black indicates no significant change in gene expression.

Terpenoids are biosynthesized through the MEP and MVA pathways. To further investigate the associations between differential metabolic networks involved in terpenoid biosynthesis, we screened and characterized the expression profiles of DEGs and accumulation levels of DAMs in the sesquiterpenoid biosynthesis pathway of *A. chinensis* under drought stress. Variations in the abundance of genes and metabolites enriched in the sesquiterpenoid and triterpenoid biosynthesis pathways exhibited distinct differences among different drought stress groups (**Figure 8**). A total of 38 single genes encoding 9 known enzymes in the MVA pathway, 19 single genes encoding 7 known enzymes in the MEP pathway, and 41 single genes encoding downstream enzymes related to terpenoid biosynthesis were screened. Among these, up to 12 single-copy genes encoded (-)-Germacrene D synthase (GERD, EC:4.2.3.75), *TRINITY_DN85559_c0_g2* and *TRINITY_DN3978_c0_g1* were most significantly up-regulated, while *TRINITY_DN16077_c0_g1* and *TRINITY_DN36616_c0_g1* were most significantly down-regulated. Subsequently, 9 single-copy genes encoded Geranylgeranyl diphosphate synthase (GPPS, EC:2.5.1.29), *TRINITY_DN37656_c0_g1* showed the highest up-regulation, and *TRINITY_DN5499_c0_g1* exhibited the most prominent down-regulation. Three DAMs were screened out, namely Germacra-1(10),4,11(13)-trien-12-ol, Nivalenol, and β-Amyrin. Notably, Germacra-1(10),4,11(13)-trien-12-ol was up-regulated under LDS and MDS but down-regulated under SDS, indicating it may serve as a critical metabolite of *A. chinensis* in responding to drought stress.

## Discussion

A. *chinensis* is an important medicinal plant in traditional Chinese medicine, its rhizomes are mainly used for the treatment of conditions such as damp obstruction in the middle energizer, epigastric distension and fullness, diarrhea, edema, and wind-damp bi syndrome. In modern clinical practice, with the in-depth research on its active ingredients, the clinical application scope of *A. chinensis* has been further expanded. Its extracts exert anti-gastritis activity (Hossen et al., 2019). Meanwhile, they can be used as potential antibacterial agents against *Salmonella typhimurium* (Gao et al., 2022) and exert a synergistic anti-gastric cancer effect in combination with oxaliplatin (Liang et al., 2024).Three years is generally considered the optimal age for harvesting rhizomes (Liu et al., 2021**;**Chen et al., 2024), and, according to the theory of “temporal pharmacognosy”, mid-to-late October (i.e., the fruiting stage) is the optimal time to harvest (Li Y. et al., 2024). The theory of temporal pharmacognosy is an applied discipline that studies the general temporal patterns of the accumulation of active ingredients, harvest period, storage of medicinal materials, pharmacological effects, and administration of crude drug sources, ensuring their quality and enhancing their efficacy (Kang et al., 1994). The harvesting time of crude drugs is directly related to clinical efficacy. As recorded in *Yong Yao Fa Xiang* (Pharmacological Images of Medicinal Use) by Wang Haogu in the Yuan Dynasty: “*Roots, leaves, flowers, and fruits must be harvested at the right time; missing the proper time will result in incomplete nature and flavor*” (Zhou and Duan, 2013). In other words, crude drugs harvested at the optimal period possess high quality. Therefore, in this study, two-year-old *A. chinensis* seedlings were selected for a one-year drought stress treatment. Drought stress was initiated at the seedling stage, followed by the vegetative growth stage and flowering stage, with sampling conducted at the final fruiting stage. This design aims to explore the effects of long-term drought stress on the sesquiterpenoid components of *A. chinensis*.

The medicinal part of *A. chinensis* is its rhizome, and sesquiterpenoids are the main secondary metabolites accumulated in it. Atractylodin, β-eudesmol, atractylenolide I, and other compounds are the major bioactive components. To date, approximately 80 sesquiterpenoid compounds have been identified and detected (Kim and Kim, 2022), which are mainly classified into three types: azadirane-type, guaiane-type, and menthane-type (Zhang C.C. et al., 2025). The skeleton of sesquiterpenoids is composed of three isoprene units, containing 15 carbon atoms (Umlauf et al., 2004). The results showed that, the contents of atractylodin, β-eudesmol, atractylenolide I in *A. chinensis* increased under LDS and MDS, while their contents decreased under SDS. This indicates that drought stress activates the regulatory mechanism of secondary metabolism and modulates the expression of relevant stress-protective genes to adapt to or resist drought stress. This is consistent with previous reports that secondary metabolites increase under light and moderate drought conditions but decrease under severe drought stress (Huang et al., 2023**;**Li J. et al., 2024).

When plants are subjected to stress and suffer certain damage, they will activate the increased expression of key enzyme genes in secondary metabolism, leading to increased activity of such enzymes and thus promoting the accumulation of corresponding specific secondary metabolites (Wang et al., 2022). Relevant studies have clarified the functions and molecular mechanisms of key genes related to drought stress responses by exploring gene expression levels, molecular biological functions, and signal regulatory pathways (Chen et al., 2023**;**Zhang Y. et al., 2025**;**Liu et al., 2024). Sesquiterpenoids are widely distributed secondary metabolites with diverse functions, which are derived from the MVA pathway and MEP pathway in plants (Zhao et al., 2024**;**Wang et al., 2024). The MVA pathway is the main biosynthetic pathway for terpenoids in *A. chinensis* in response to drought stress. Key enzymes in the MVA pathway include acetyl-CoA C-acetyltransferase (AACT, EC:2.3.1.9), 3-hydroxy-3-methylglutaryl-CoA reductase (HMGR, EC:1.1.1.34), and 3-hydroxy-3-methylglutaryl-CoA synthase (HMGS, EC:2.3.3.10), which are important rate-determining steps in the terpenoid biosynthetic pathway (Sun et al., 2024**;**Li X. et al., 2024). Key enzymes in the MEP pathway include 1-deoxy-D-xylulose-5-phosphate reductoisomerase (DXR, EC:1.1.1.267), 1-deoxy-D-xylulose-5-phosphate synthase (DXS, EC:2.2.1.7), and 2-C-methyl-D-erythritol 4-phosphate cytidylyltransferase (MCT, EC:2.7.7.60) (Zhang C. et al., 2025; Huang et al., 2021).

We acquired transcriptomic and metabolomic datasets, followed by integrated multi-omics analysis, to identify additional key genes and critical metabolites associated with the sesquiterpenoid biosynthesis pathway of *A. chinensis* under drought stress. Via this integrated approach, we detected 38 single-copy genes encoding 9 core key enzymes (including AACT, HMGS, and HMGR) in the MVA pathway, 19 single-copy genes encoding 7 core key enzymes (including DXS, DXR, and MCT) in the MEP pathway, and 41 single-copy genes encoding downstream enzymes related to terpenoid biosynthesis. In total, 98 single-copy genes were involved in sesquiterpenoid biosynthesis in *A. chinensis* under drought stress. Additionally, we observed that Germacra-1(10),4,11(13)-trien-12-ol was up-regulated under LDS and MDS but down-regulated under SDS—this expression pattern aligned with the content fluctuations of sesquiterpenoid constituents (atractylodin, β-eudesmol, and atractylenolide I) under drought stress, indicating it functions as a critical metabolite. Correlation analyses between DEGs and DAMs demonstrated that *TRINITY_DN12874_c1_g1*, *TRINITY_DN114406_c0_g1*, *TRINITY_DN2331_c0_g2*, *TRINITY_DN7401_c0_g1*, and *TRINITY_DN11676_c0_g1* exert positive regulatory effects on the metabolite Germacra-1(10),4,11(13)-trien-12-ol, thus classifying these five genes as key genes.

Homologous sequence alignment analysis via NCBI BLASTn revealed that *TRINITY_DN12874_c1_g1* shared a high homology with *FPPS* (similarity > 90%), *TRINITY_DN114406_c0_g1* with Sesquiterpene Synthase (*STPS*, similarity > 88%), *TRINITY_DN2331_c0_g2* with *HMGR* (similarity > 92%), TRINITY_DN7401_c0_g1 with Mevalonate Kinase (*MK*, similarity > 90%), and *TRINITY_DN11676_c0_g1* with Isopentenyl Diphosphate Isomerase (*IDI*, similarity > 89%). *FPPS* might contribute to enhancing drought tolerance (Pan et al., 2017). STPS belongs to the terpene synthase (TPS) family, TPS is related to abiotic stress and plant development (Zhan et al., 2022). Temperature stress predominantly up-regulated related genes such as IDI,HMGR,FPPS, which demonstrated strong connectivity and correlations with saiko saponins content (Gu et al., 2025). MK is a member of the GHMP kinase gene family, and plays a pivotal role in regulating plant growth and development as well as mediating various stress responses (Yang et al., 2025).

In conclusion, 98 genes are involved in the biosynthesis of sesquiterpenoids in *A. chinensis* under drought stress. It is inferred that the genes (*TRINITY_DN12874_c1_g1*, *TRINITY_DN114406_c0_g1*, *TRINITY_DN2331_c0_g2*, *TRINITY_DN7401_c0_g1*, and *TRINITY_DN11676_c0_g1*) and the metabolite Germacra-1(10),4,11(13)-trien-12-ol are the key genes and critical metabolites for *A. chinensis* in response to drought stress.

## Conclusions

In this study, *A. chinensis* was subjected to a one-year drought stress treatment. Drought stress affected the accumulation of sesquiterpenoids in *Atractylodis Rhioma*, with LDS and MDS promoting the accumulation of atractylodin, β-eudesmol and atractylenolide I. A soil water content of 35%–60% of the field capacity was conducive to achieving optimal growth and performance of *A. chinensis*. Combined with transcriptomic and metabolomic analyses, 98 genes involved in sesquiterpenoid biosynthesis were screened out. The metabolite germacra-1(10),4,11(13)-trien-12-ol was up-regulated under LDS and MDS but down-regulated under SDS, which was consistent with the changes in the contents of atractylodin, β-eudesmol and atractylenolide I after drought stress. Additionally, *TRINITY_DN12874_c1_g1*, *TRINITY_DN114406_c0_g1*, *TRINITY_DN2331_c0_g2*, *TRINITY_DN7401_c0_g1* and *TRINITY_DN11676_c0_g1* showed a positive correlation with the synthesis of the sesquiterpenoid metabolite germacra-1(10),4,11(13)-trien-12-ol. This study deepens our understanding of the molecular mechanism underlying drought stress-mediated regulation of sesquiterpenoid synthesis, and it is of great significance for improving the quality of *Atractylodis Rhioma*.

## Data Availability

The original contributions presented in the study are included in the article/**Supplementary Material**. Further inquiries can be directed to the corresponding authors.
