# Supplementary material for: Integrated transcriptome and metabolome analysis reveal the sesquiterpenoid biosynthesis mechanism of Atractylodes chinensis under drought stress
Source: Front Plant Sci. 2026 Jan 26;16:1751860. doi: 10.3389/fpls.2025.1751860 (PMC12884327; doi:10.3389/fpls.2025.1751860)
Supplement: Supplementary file 1 [file Image1.pdf]

CK1

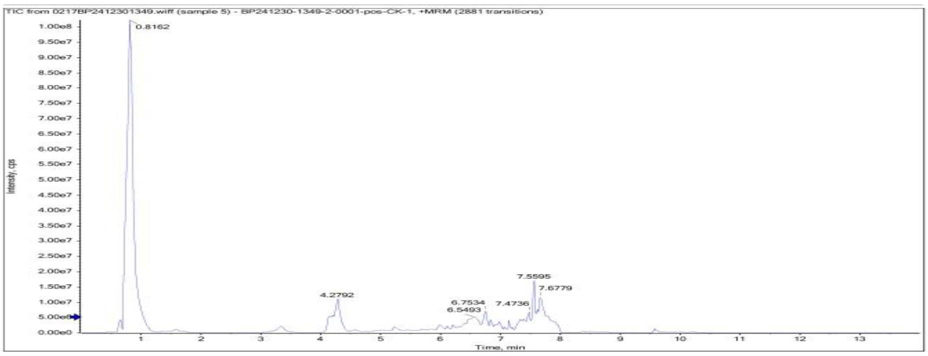

CK2

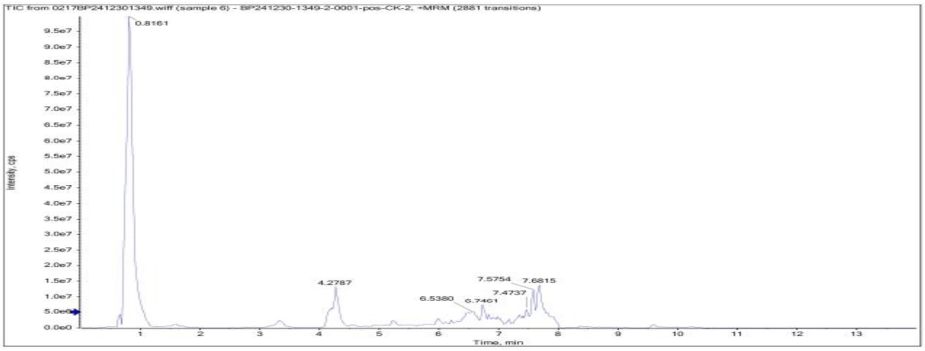

CK3

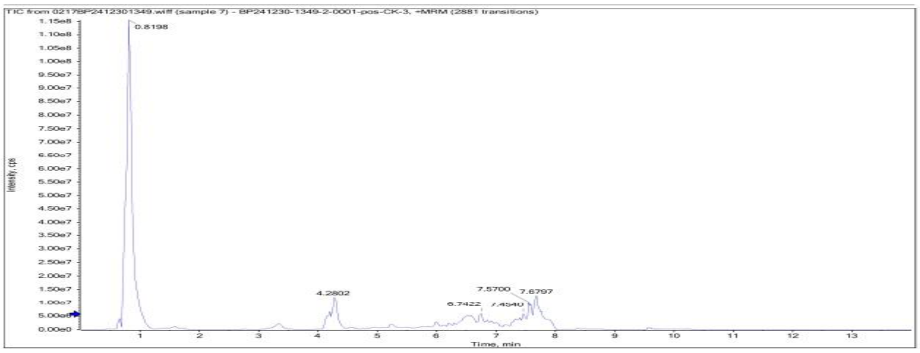

LDS1

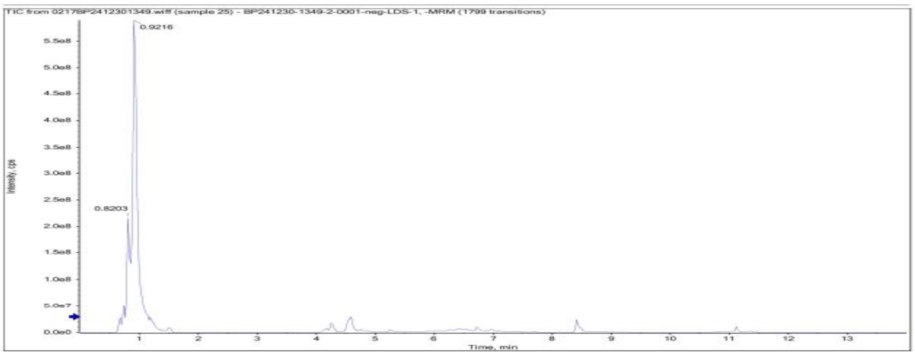

LDS2

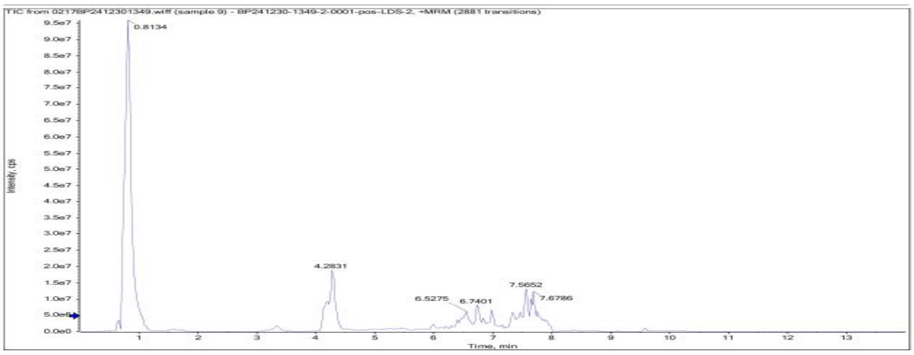

LDS3

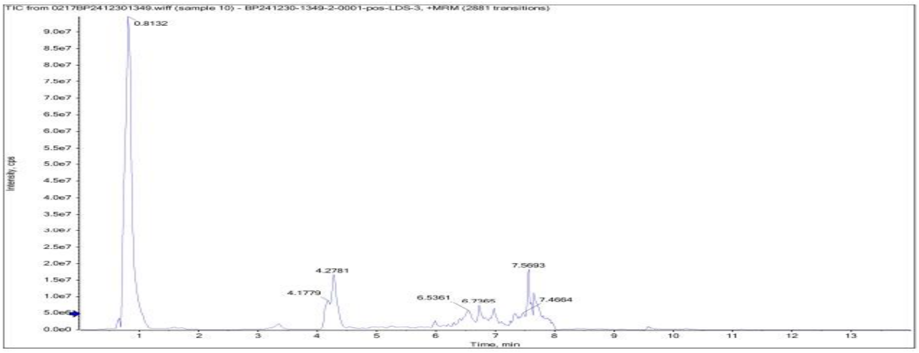

MDS1

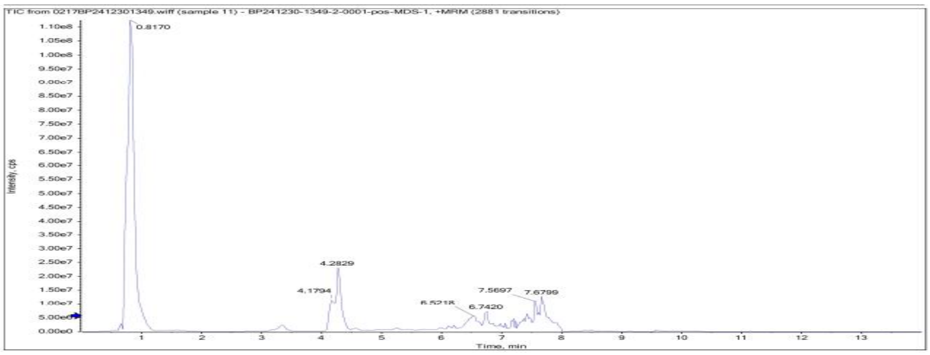

MDS2

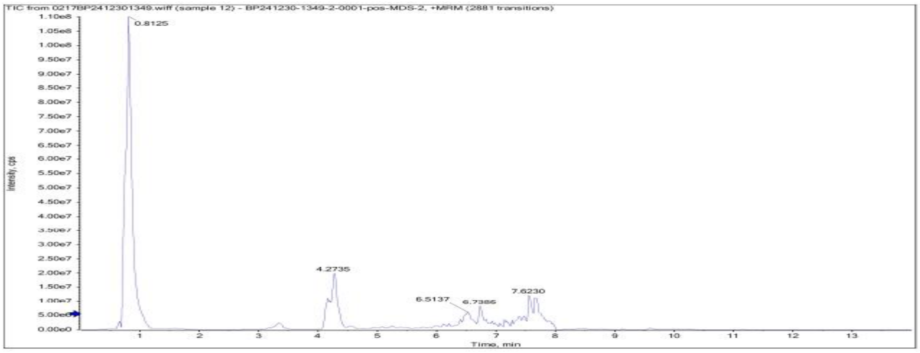

MDS3

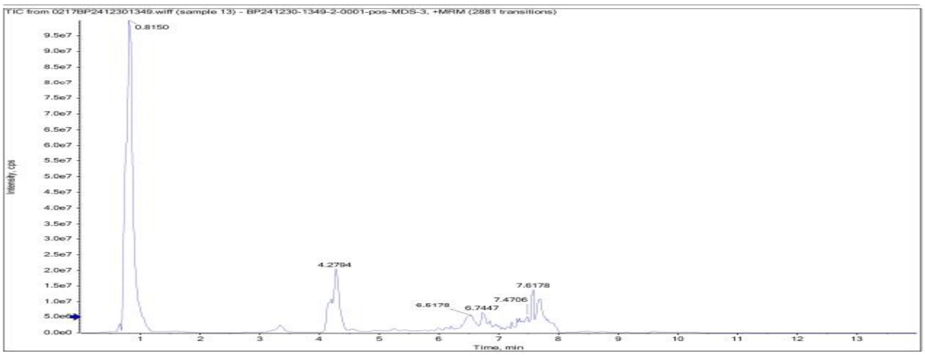

SDS1

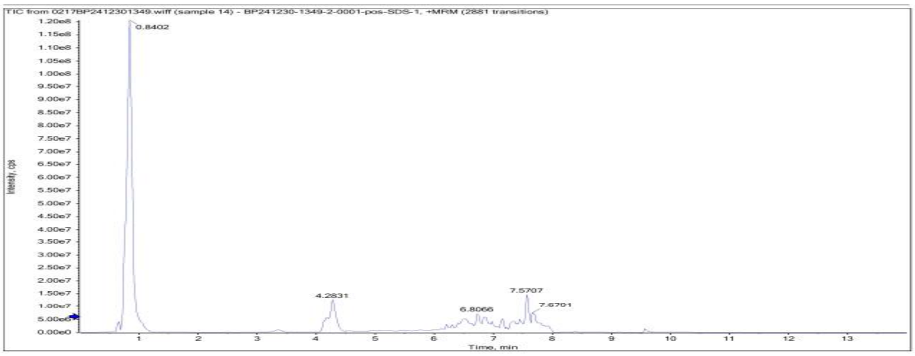

SDS2

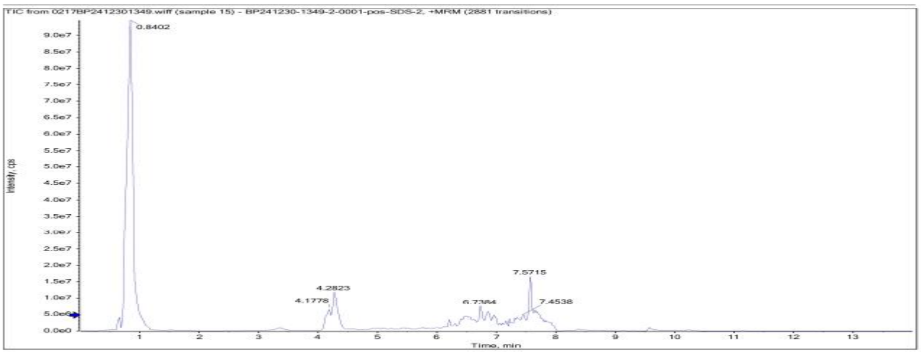

SDS3

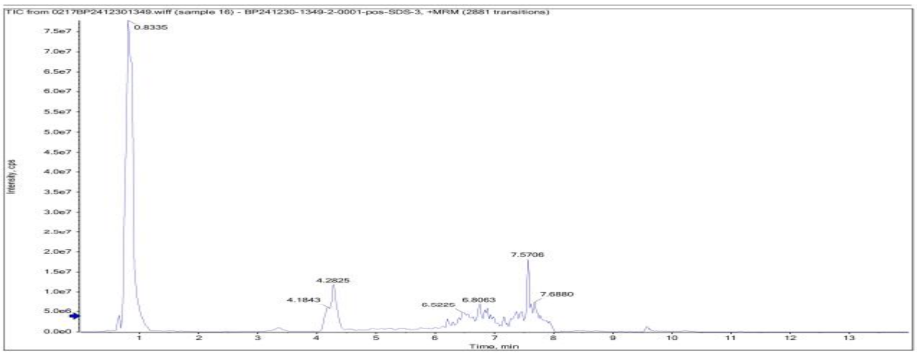

Supplementary Figure 1. UPLC-MS/MS Chromatograms of metabolites from all samples
